# Artificial Neural Network Models for Accurate Predictions of Fat-Free and Fat Masses, Using Easy-to-Measure Anthropometric Parameters

**DOI:** 10.3390/biomedicines11020489

**Published:** 2023-02-08

**Authors:** Ivona Mitu, Cristina-Daniela Dimitriu, Ovidiu Mitu, Cristina Preda, Florin Mitu, Manuela Ciocoiu

**Affiliations:** 1 Department of Morpho-Functional Sciences II, University of Medicine and Pharmacy “Grigore T. Popa”, 700115 Iasi, Romania; 2 1st Medical Department, University of Medicine and Pharmacy “Grigore T. Popa”, 700115 Iasi, Romania; 3 Department of Endocrinology, University of Medicine and Pharmacy “Grigore T. Popa”, 700115 Iasi, Romania

**Keywords:** trunk fat mass, trunk fat-free mass, lean mass, fat mass, body composition, machine learning, artificial neural network, obesity, adiposity, cardiometabolic risk

## Abstract

Abdominal fat and fat-free masses report a close association with cardiometabolic risks, therefore this specific body compartment presents more interest than whole-body masses. This research aimed to develop accurate algorithms that predict body masses and specifically trunk fat and fat-free masses from easy to measure parameters in any setting. The study included 104 apparently healthy subjects, but with a higher-than-normal percent of adiposity or waist circumference. Multiple linear regression (MLR) and artificial neural network (ANN) models were built for predicting abdominal fat and fat-free masses in patients with relatively low cardiometabolic risks. The data were divided into training, validation and test sets, and this process was repeated 20 times per each model to reduce the bias of data division on model accuracy. The best performance models used a maximum number of five anthropometric inputs, with higher R^2^ values for ANN models than for MLR models (R^2^ = 0.96–0.98 vs. R^2^ = 0.80–0.94, *p* = 0.006). The root mean square error (RMSE) for all predicted parameters was significantly lower for ANN models than for MLR models, suggesting a higher accuracy for ANN models. From all body masses predicted, trunk fat mass and fat-free mass registered the best performance with ANN, allowing a possible error of 1.84 kg for predicting the correct trunk fat mass and 1.48 kg for predicting the correct trunk fat-free mass. The developed algorithms represent cost-effective prediction tools for the most relevant adipose and lean tissues involved in the physiopathology of cardiometabolic risks.

## 1. Introduction

Over the years, segmental body masses have gained more importance in stratifying the risk of diseases associated with obesity. Fat mass and more precisely, trunk fat mass, reports relevant associations with insulin resistance and dyslipidaemia [1]. At the same time, lean mass also proves a significant relationship with cardiometabolic risks [2]. Therefore, including fat and lean masses into a patient’s profile would assure precise tools for prevention and management of obesity and associated diseases. BMI is currently used for obesity definition, and waist circumference is considered equally important and feasible for improving patient management [3]. However, BMI tends to overestimate body fat mass in individuals with a high muscle mass [4]. The main advantages of these measures are the low cost and ease of determination, while segmental body masses involve high cost and access to specific equipment.

New methods to automate the process of body composition measurements are highly explored. Research in this field showed that multivariate regression models [5,6], receiver operating characteristic curves [7] and neural networks [8] are methods implemented for the prediction of body composition. Artificial intelligence and machine-learning have made promising advances in the field of image segmentation, that could be accessible in the future in a variety of clinical and research workflows [9,10].

The goal of the study is to develop accurate algorithms that predict body masses, and specifically trunk fat and fat-free masses, from easy-to-measure parameters in any setting. The focus is predominantly geared towards abdominal masses, due to their close association with cardiometabolic risks and because most studies do not focus on a specific body compartment, but rather on the whole body. To validate the best approach, we aim to compare linear regression models with neural networks that can capture non-linear relationships between variables.

## 2. Materials and Methods

This study was conducted over a period of 2 years (2020–2022) and included 104 apparently healthy subjects, but with a higher-than-normal percent of adiposity or waist circumference. The selected participants had no antecedent atherosclerotic acute event and no known chronic disease, or had not followed treatment in the last 6 months. Pregnant women were excluded from the study, since all patients underwent dual X-ray absorptiometry (DEXA) investigation. For every participant, anthropometric and demographic data were collected. The study was approved by the University of Medicine and Pharmacy “Gr. T. Popa” Ethics Committee, number 1/27 July 2020 and all participants signed an informed consent form before entering the study.

### 2.1. Baseline Characteristics of the Study Population

Anthropometric measures were assessed by the same specialised medical staff during the entire period of the study, using the same techniques and instruments. All investigations were performed after a 12 h overnight fast, including no prior consumption of liquids in the respective morning. The DEXA examinations were assessed with a Hologic QDR Delphi A fan-beam densitometer (Hologic Inc., Malborough, MA, USA). Height was measured with a stadiometer, waist circumference (WC) and hip circumference (HC) with a flexible tape, abdominal and tricipital skinfold with a Holtain-type caliper. Tricipital skinfold was assessed halfway between the acromion process and olecranon process, and abdominal skinfold at 5 cm lateral of the umbilicus [11]. Waist circumference was measured between the last rib and the iliac crest at its smallest perimeter, and hip circumference at the greater trochanter level [12].

### 2.2. Development of Fat Mass and Fat-Free Mass Estimation Models

This research developed prediction models for body composition parameters that have the highest influence on metabolic syndrome (MetS) prevalence. To achieve the best performance, a comparison was performed between multiple linear regression (MLR) and artificial neural network (ANN) models. The goodness of fit of the models was evaluated using the mean squared error (MSE) and the coefficient of determination (R^2^). For the purpose of comparison, the same formula was used to calculate the error, both in SPSS and in MATLAB (Equation (1)):

(1) $$\mathrm{MSE}=\frac{1}{n}\overset{n}{\underset{i=1}{\sum }}{\left(Y_{observed}-Y_{predicted}\right)}^{2}$$

#### 2.2.1. MLR Models

MLR models are easy to implement and imply low computational power. In this analysis, we built MLR models for predicting the dependent variables Total Fat Mass (kg), Total Fat-free Mass (kg), Trunk Total Mass (kg), Trunk Fat Mass (kg), Trunk Fat-Free Mass (kg). The initial independent variables used for estimation were seven continuous demographic and anthropometric parameters, that are easily assessed by trained personnel in any medical facility.

#### 2.2.2. ANN Models

Multilayer, perceptron, feed-forward neural network models were used for predicting the same dependent variables as mentioned for the MLR models. The units in the model are trained with the Levenberg–Marquardt backpropagation learning algorithm and the prediction is performed by the output layer. The input layer consists of continuous demographic and anthropometric variables, and the arbitrary number of units in the hidden layer between the input and output represent the true computational engine of the model [13]. Based on the Universal Approximation Theorem, “neural networks with a single hidden layer can be used to approximate any continuous function to any desired precision” [14]. Therefore, we chose to work with only one hidden layer. The initialization function was randomized weights. The tansig (hyperbolic tangent sigmoid) activation function was used for the units in the hidden layer, and the purelin (linear) activation function for the output layer. The maximum number of iterations for the algorithm was set to 1000.

The input parameters were included based on human logic and ease of use in clinical settings. Afterwards, a forward-feature selection method was conducted to find the best model. The data were randomly divided into training set (75%), validation set (15%) and test set (15%), and each time there was a network computed. Twenty particular cases associated with the number of units in the hidden layer were considered, from 1 to 20. The performance did not improve after reaching 20 cases for each parameter estimated, therefore we concluded that this number is representative for reaching the best performance. Each model was trained, validated and tested in a sequence of 20 iterations. For each iteration the mean squared error (MSE) for the entire set (Equation (1)) was calculated, and after all iterations the averaged MSE_set_ was attributed to that specific model with the respective number of units in the hidden layer (Equation (2)).

(2) $$({\mathrm{MSE}}_{\mathrm{set}})=0.7\times {\mathrm{MSE}}_{\mathrm{tr}}+0.15\times {\mathrm{MSE}}_{\mathrm{val}}+0.15\times {\mathrm{MSE}}_{\mathrm{test}}$$

**Figure 1:**
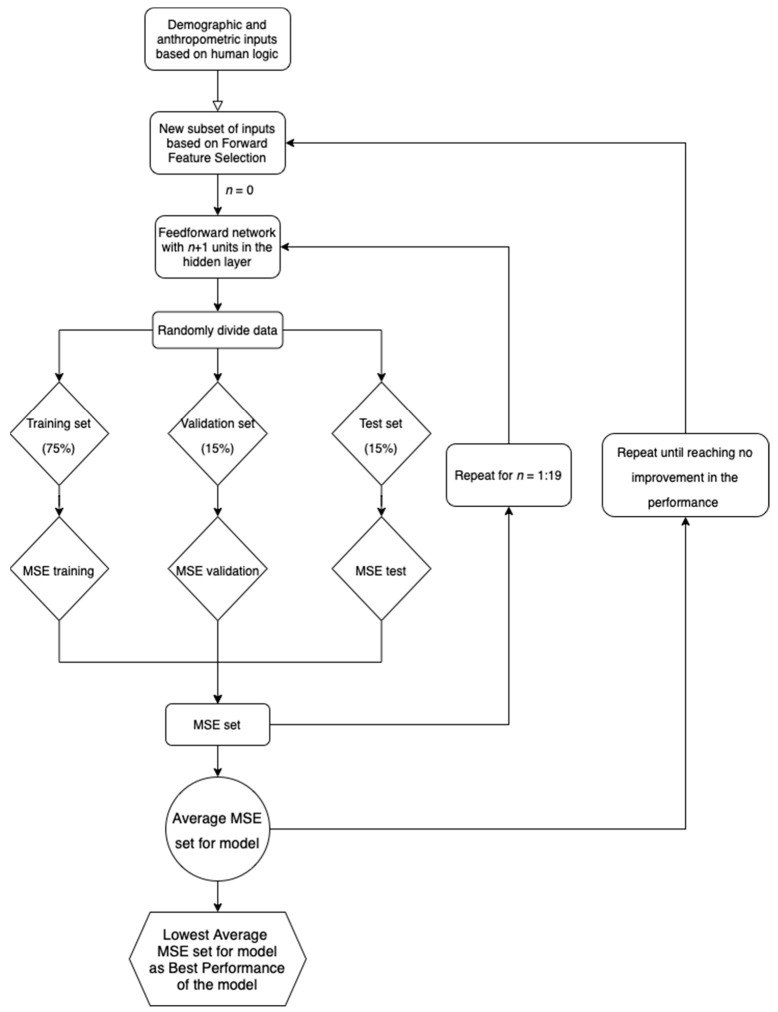
Flowchart for the algorithm used to choose the best models.

The 20 iterations per case were necessary as to evaluate the effect of data division on the goodness of fit of the model. The goal is to have similar data sets for the model, but chosen randomly, as in real life. The lowest averaged MSE of all 20 confirmed the number of units in the hidden layer needed to identify the best performance of the model. It also lowered the risk of bias in that perspective. From the 20 cases, the one with the lowest MSE was selected as the model with the best performance. The root mean square error (RMSE) was calculated for the chosen model. A simplified version of the methods used for choosing the best models is presented in Figure 1.

### 2.3. Statistical Analysis

There were no missing data, therefore no data substitution algorithm was necessary. All variables were analysed using Microsoft Excel version 16.64 (Microsoft Corporation, Redmond, WA, USA), SPSS version 23.0 (IBM Corporation, Armonk, NY, USA) and MATLAB R2021b (The MathWorks Inc., Natick, MA, USA).

MLR models were computed in SPSS, using stepwise regression. This method excluded variables that assumed multicollinearity. ANN models were built with Neural Network Toolbox in MATLAB. For ease of use, an in-house function was constructed to save the network with the best performance per each input in the hidden layer.

All continuous variables were tested for normality with Kolmogorov–Smirnov test. The normally distributed data were reported as the means ± standard deviations (SD), and the non-normally distributed data were reported as the median and quartiles. Categorical variables were expressed as frequencies (percentages). Linear regression generated the R^2^ and MSE values. The RMSE and R^2^ values for the chosen models were normally distributed, therefore an independent sample *t*-test was used to compare these errors and R^2^ between the MLR and ANN models.

For assessing multicollinearity, the Pearson correlation was used for all data. Results were considered statistically significant if *p* < 0.05.

## 3. Results

All the demographic and anthropometric measures are reported in Table 1, together with total fat and fat-free masses for the trunk region and for the whole body. The fat-free mass parameter includes lean and bone mineral content for that specific region, with the amendment that fat-free mass measured by DEXA is smaller than the classical concept of lean body mass presented in the literature for the first time eight decades ago, which also includes essential fat [15]. The confusion of terminology in the literature leads to improper comparisons between studies and it should be avoided by clearly stating the definition of lean mass in the respective study.

**Figure 2:**
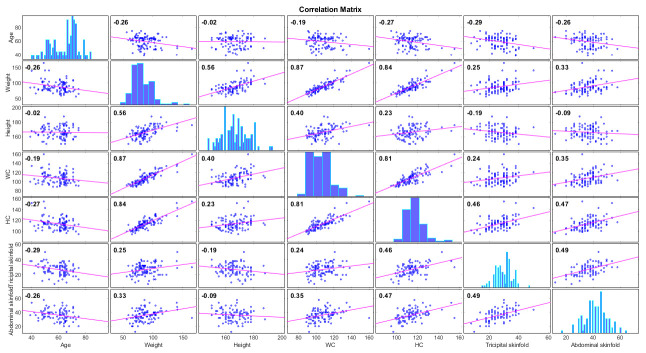
Correlation matrix for variables considered in the model development.

The initial input variables included in the algorithm were age, weight, height, waist circumference, hip circumference, tricipital skinfold and abdominal skinfold. Pearson correlation was performed to exclude high intercorrelation (≥0.9) between variables. The r coefficient is written on each plot, with a value ranging from –0.02 to 0.872, therefore no high intercorrelation was detected (Figure 2).

The initial attempt was to obtain prediction algorithms for percentages of fat and fat-free masses, as these are proven to be more relevant for estimating the cardiovascular risk of a patient. These initial results yielded models (both MLR and ANN) with poor performance, guiding us to estimating the fat and fat-free mass in kilograms that would allow afterwards the calculus of percentages.

The stepwise method for MLR identified the independent variables with the highest level of prediction on the outcome and the best performances (Table 2). After performing forward-feature selection for the machine-learning algorithm, the best performances of the ANN models for each parameter were assessed (Table 3).

**Figure 3:**
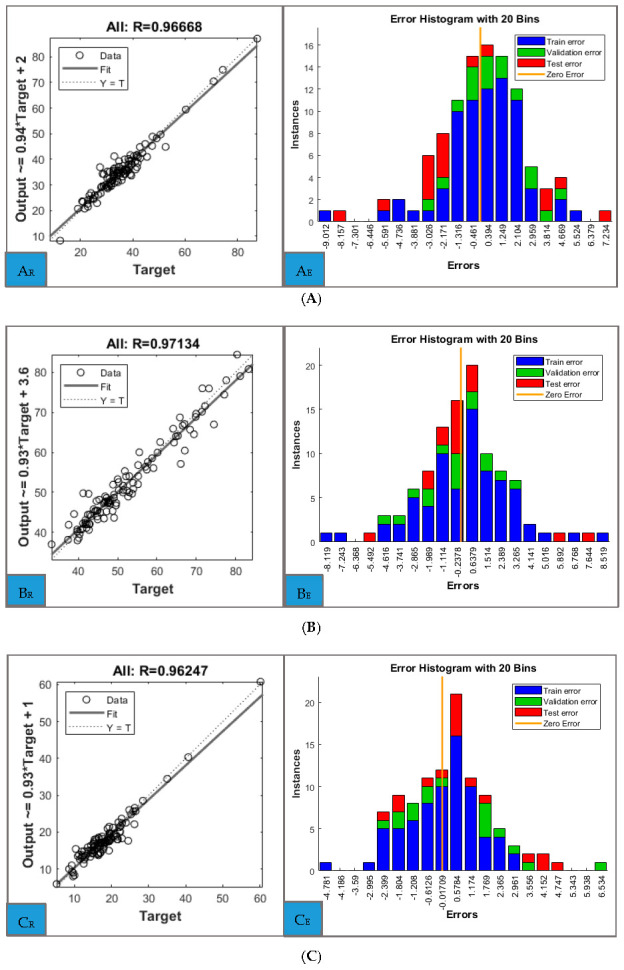

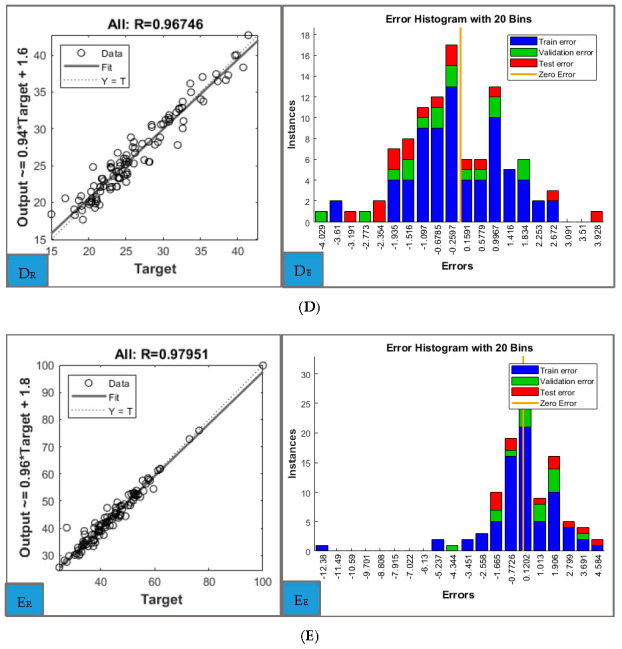
Regression plots (left) and error histograms (right) for: (**A**) Total fat (kg), (**B**) Total Fat-free (kg), (**C**) Trunk fat (kg), (**D**) Trunk Fat-free (kg), (**E**) Trunk total (kg).

The regression plots for the entire set of data for each predicted parameter can be analysed in Figure 3. Beside high coefficients of determination which confirm the accuracy of the model, close examination of the errors of the ANN models is needed. These are presented separately for the training, validation and test sets for each predictor (Figure 3). The individual regression plots for training, validation and test sets for each parameter are included in the Supplementary Materials (Figures S1–S10), together with performance and training state plots.

**Figure 4:**
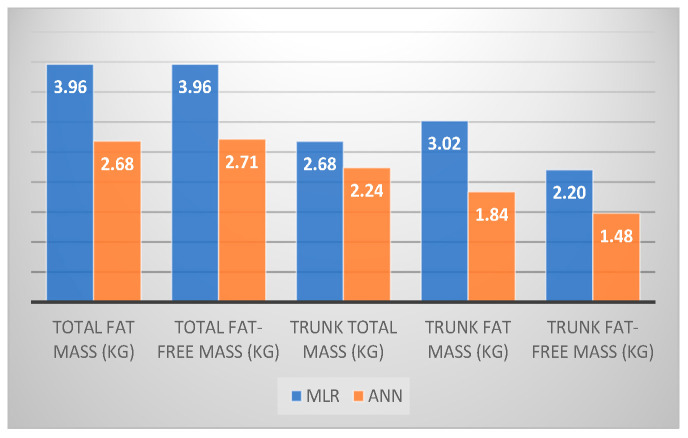
Comparison between RMSE values for MLR and ANN models.

The R^2^ values reported for the ANN models are significantly higher than the ones for the MLR models (*p* = 0.006), suggesting a good prediction of the outcome. In Figure 4, we plot the RMSE values for each parameter according to its own best performance model. RMSE values define the mean of error in a more practical way, concluding that for ANN models the mean error will be for all parameters between 1.48–2.71 kg vs. 2.20–3.96 kg for MLR models (*p* = 0.05).

## 4. Discussion

Our previous research on the same cohort reported fat and fat-free percentage high sensitivity scores regarding the prevalence of MetS, concluding that body composition holds an important place in prevention and management programs of MetS [16]. These are measurements provided by DEXA, which is an accurate and validated method in evaluating body composition [17,18,19]. The high cost of this investigation justifies our approach to predict these parameters from simple measurements that can be performed in any medical clinic. Percentages of adipose tissue or of lean mass are preferable to masses in kg, since they offer a perspective of the patient’s body composition and they are more precise in estimating the prevalence of MetS [16]. Since the models for estimating percentages presented low performance, all efforts were redirected towards estimating all the necessary values for calculating the percentages.

Extended studies have shown that body masses establish a more accurate relationship with mortality than BMI, in large populations [20,21,22]. BMI does not distinguish fat mass from lean mass, thus defining optimal thresholds for this measurement is not enough [23]. Furthermore, a thorough risk stratification can be obtained by separating visceral fat from subcutaneous fat and identifying sick fat (adiposopathy). This ratio is in favour of visceral fat in cardiovascular diseases [24,25], moreover that patients undergoing lifestyle interventions (diet and exercise, weight-loss medication, bariatric surgery) do not preferentially loose one type of tissue over the other [26]. The cardiovascular health depends on the type of adiposity, its inflammation level, its location and function [27,28]. Extended studies stress the importance of correctly identifying body masses in order to predict the cardiometabolic risk the patient is facing more accurately.

The proposed prediction models are specific for patients that are apparently healthy, with no previous diagnosis of MetS, but with a higher-than-normal percentage of adipose tissue or waist circumference. The metabolic profile of patients is presented elsewhere [16]. There are several research studies that report models for estimating fat mass, but even though they include a high number of participants, most of these studies had not targeted specific subpopulations as to reduce bias [29,30,31]. A retrospective study on 14,065 individuals proposes a multiple linear regression model with a high goodness of fit for estimating lean and fat mass, suggesting a generalizability of the model [32]. This study proposes a model for patients with a higher-than-normal fat mass that are at borderline or have just been diagnosed with MetS. The strength of the study is that the inclusion criteria for participants best fits the purpose of estimating body masses in patients with no, or relatively low, cardiometabolic risks. The proposed algorithm has a low bias, argued by no prior medication or no diagnosed chronic diseases. Furthermore, participants with MetS are at an initial stage of the pathology, thus the cohort is also characteristic for those individuals that cross the fine line between healthy and unhealthy.

The ANN models resulted are superior to the MLR ones, therefore machine learning algorithms represent a better choice for capturing the relationship between body masses and anthropometric measurements on a cohort similar to the one analysed. More advanced work underlines the idea that hybrid models, like the one proposed by Hussain S.A. et al., based on support vector regression and emotional artificial neural networks, provide superior results to other machine-learning models [33]. A recent study on 20,137 subjects reported similar results for lean and fat masses, with higher R^2^ and lower standard error of the estimate values for ANN than for MLR, while using 13 demographic and anthropometric measures as predictors [34]. The research presented in this paper proposes simpler models with a maximum number of five anthropometric inputs, also with high R^2^ values, suggesting that these models explain the variability of a large portion of the dataset. The error is higher than the abovementioned study, mainly due to the small cohort and possibly to the particularity of the population selected, preMets and MetS niched. Another strength is that test data were included to confirm the good performance of the model, beside the validation set that ensured no overfitting.

Most studies estimate total fat and lean masses, and not masses specific to certain areas of the body. This study answers the necessity of narrowing down to abdominal mass, since adiposity at this level is the most important causative factor for cardiometabolic diseases. On the other hand, patients with low lean mass and high fat mass associate with a higher mortality than patients with normal lean mass and high fat mass [35]. Considering this and the fact that in our previous study trunk fat-free mass was reported as the body composition parameter with the best predictive power for the prevalence of MetS, its value is important specifically in stratifying MetS risks [16]. From all body masses predicted, trunk fat-free mass registered the best performance, with an R^2^ value per entire set of 0.98, MSE of 2.19 kg and RMSE of 1.48 kg.

The algorithms developed on the population included in the study provide similar results to the ones for the white population. The multi-ethnic research on the same subject reported similar values for errors according to the ANN models: approximately 2 kg for training set for total adipose tissue mass in the white population vs. 2.35 kg in this study. The error values for the MLR model were lower in the multi-ethnic study: approximately 2.45 kg vs. 3.96 kg in this study. Although standard error of the estimate was used to report the goodness of fit of the models, RMSE uses almost the same formula, therefore, the values can be safely compared [34].

Continuous addition of participants with the same characteristics in the training and validation set will allow the error to decrease and, therefore, further improve the accuracy of the models developed in this study. A variability in DEXA devices concerning calibration procedure, variations in photon source intensities or the formulas implemented in the software [36], are also acknowledged. The specific and well-chosen inclusion criteria, together with providing a test set for confirming the accuracy of the prediction algorithms, provide low bias and validated results.

## 5. Conclusions

This study proposes alternative algorithms to determining total fat-free and fat masses and more specifically, trunk fat-free and fat masses. These high-cost measurements normally require access to hospital equipment and a complex set-up, justifying our approach for estimating them from easily obtainable anthropometric inputs. The ANN models showed a better and more statistically significant performance than MLR ones, with a lower error for all predicted parameters. Trunk fat-free and fat masses presented the models with the best accuracy, supporting the research for prediction tools of the most relevant adipose and lean tissues involved in the physiopathology of cardiometabolic risks. These algorithms provide a resource for better assessing cardiovascular risk in patients, developing scores for obesity, improving the management of weight-loss and prevention programs.

## Figures and Tables

**Table 1:** Baseline characteristics of the study population.

Study Population (*n* = 104)				
**Parameter**	Percentage (number of subjects)			
Gender				
Female	74.04% (77)			
Male	25.96% (27)			
Residence				
Urban	63.46% (66)			
Rural	36.54% (38)			
Normally distributed continuous variables				
	Mean ± SD	95% CI		Kolmogorov–Smirnov *p* value
		Lower bound	Upper bound	
Height (cm)	166.13 ± 7.83	164.61	167.66	0.144
Tricipital skinfold (mm)	27.06 ± 7.43	25.61	28.50	0.175
Abdominal skinfold (mm)	36.22 ± 8.17	34.63	37.81	0.200
Trunk fat %	40.30 ± 6.42	39.05	41.55	0.200
Trunk fat-free %	59.70 ± 6.42	58.45	60.95	0.200
Non-normally distributed continuous variables				
	Median	Q1	Q3	Kolmogorov–Smirnov *p* value
Age (years)	62	53	65	<0.001
Weight (kg)	84.42	76.10	98.65	0.001
WC (cm)	106	99	115	0.011
HC (cm)	113	106.25	119	0.013
BMI (kg/m^2^)	30.99	28.57	34.39	0.006
Total fat mass (kg)	34.16	30.14	39.36	<0.001
Total fat-free mass (kg)	49.07	43.97	59.04	<0.001
Trunk fat mass (kg)	17.32	14.78	20.54	<0.001
Trunk fat-free mass (kg)	24.96	21.59	30.24	<0.001
Total fat %	41.10	36.32	44.27	0.034
Total fat-free %	58.90	55.72	63.67	0.034

Note: WC = waist circumference, HP = hip circumference.

**Table 2:** Best performance MLR models for fat and fat-free masses.

Parameter Predicted	Model	β	*p*	F (df1, df2)	R^2^	*p*	MSE
Total Fat Mass (kg)	Intercept	18.599	0.236	117.71 (5, 98)	0.86	<0.001	15.71
	HC	0.389	<0.001				
	Weight	0.442	<0.001				
	Height	–0.322	<0.001				
	Tricipital skinfold	0.203	0.003				
	WC	–0.172	0.015				
Total Fat-Free Mass (kg)	Intercept	–18.675	0.234	141.901 (5, 98)	0.88	<0.001	15.70
	Weight	0.557	<0.001				
	HC	–0.389	<0.001				
	Height	0.322	<0.001				
	Tricipital skinfold	–0.202	0.003				
	WC	0.173	0.014				
Trunk Total Mass (kg)	Intercept	0.552	0.947	399.986 (4, 99)	0.94	<0.001	7.16
	Weight	0.514	<0.001				
	WC	0.183	<0.001				
	Tricipital skinfold	–0.123	0.004				
	Height	–0.108	0.024				
Trunk Fat Mass (kg)	Intercept	16.720	0.145	131.077 (3, 100)	0.80	<0.001	9.11
	Weight	0.303	<0.001				
	Height	–0.243	<0.001				
	HC	0.132	0.042				
Trunk Fat-Free Mass (kg)	Intercept	–6.198	0.477	116.878 (5, 98)	0.86	<0.001	4.85
	Weight	0.269	<0.001				
	HC	–0.196	<0.001				
	WC	0.138	0.001				
	Height	0.112	0.009				
	Tricipital skinfold	–0.088	0.018				

Note: MSE = mean square error.

**Table 3:** Best performance ANN models for fat and fat-free masses.

Units in Hidden Layer	MSE Training Set	MSE Validation Set	MSE Test Set	MSE Entire Set	R^2^ Training Set	R^2^ Validation Set	R^2^ Test Set	R^2^ Entire Set	Best Epoch
ANN 1 for Total Fat mass (kg)									
8	5.51	5.26	16.88	7.18	0.98	0.97	0.85	0.97	23
ANN 2 for Total Fat-Free mass (kg)									
3	7.78	4.56	8.20	7.36	0.97	0.98	0.97	0.97	10
ANN 3 for Trunk Total mass (kg)									
7	5.54	3.75	3.78	5.01	0.98	0.97	0.98	0.98	7
ANN 4 for Trunk Fat mass (kg)									
6	2.26	6.49	5.42	3.37	0.96	0.98	0.93	0.96	16
ANN 5 for Trunk Fat-Free mass (kg)									
6	1.71	2.76	3.86	2.19	0.98	0.93	0.94	0.97	8

Note: ANN 1: weight, WC, HC, height; ANN 2: weight, WC, HC, height, abdominal skinfold; ANN 3: weight, WC, HC, height; ANN 4: weight, WC, height, tricipital skinfold; ANN 5: weight, WC, HC, height, tricipital skinfold.

## Data Availability

Data supporting reported results are available from the corresponding authors. Data are not publicly available due to privacy.

## Supplementary Materials

The following supporting information can be downloaded at: https://www.mdpi.com/article/10.3390/biomedicines11020489/s1, Figure S1: Regression plots for Total fat mass (ANN model); Figure S2: Performance plot (left) and training state plot (right) for Total fat mass (ANN model); Figure S3: Regression plots for Total fat-free mass (ANN model); Figure S4. Performance plot (left) and training state plot (right) for Total fat-free mass (ANN model); Figure S5. Regression plots for Trunk fat mass (ANN model); Figure S6. Performance plot (left) and training state plot (right) for Trunk fat mass (ANN model); Figure S7. Regression plots for Trunk fat-free mass (ANN model); Figure S8. Performance plot (left) and training state plot (right) for Trunk fat-free mass (ANN model); Figure S9. Regression plots for Trunk total mass (ANN model); Figure S10. Performance plot (left) and training state plot (right) for Trunk total mass (ANN model).
