# Supplementary material for: Artificial Neural Network Models for Accurate Predictions of Fat-Free and Fat Masses, Using Easy-to-Measure Anthropometric Parameters
Source: Biomedicines. 2023 Feb 8;11(2):489. doi: 10.3390/biomedicines11020489 (PMC9953292; doi:10.3390/biomedicines11020489)
Supplement: Supplementary file 1 [file biomedicines-11-00489-s001.zip › biomedicines-2193756-supplementary.pdf]

Regression, performance and training state plots for ANN models

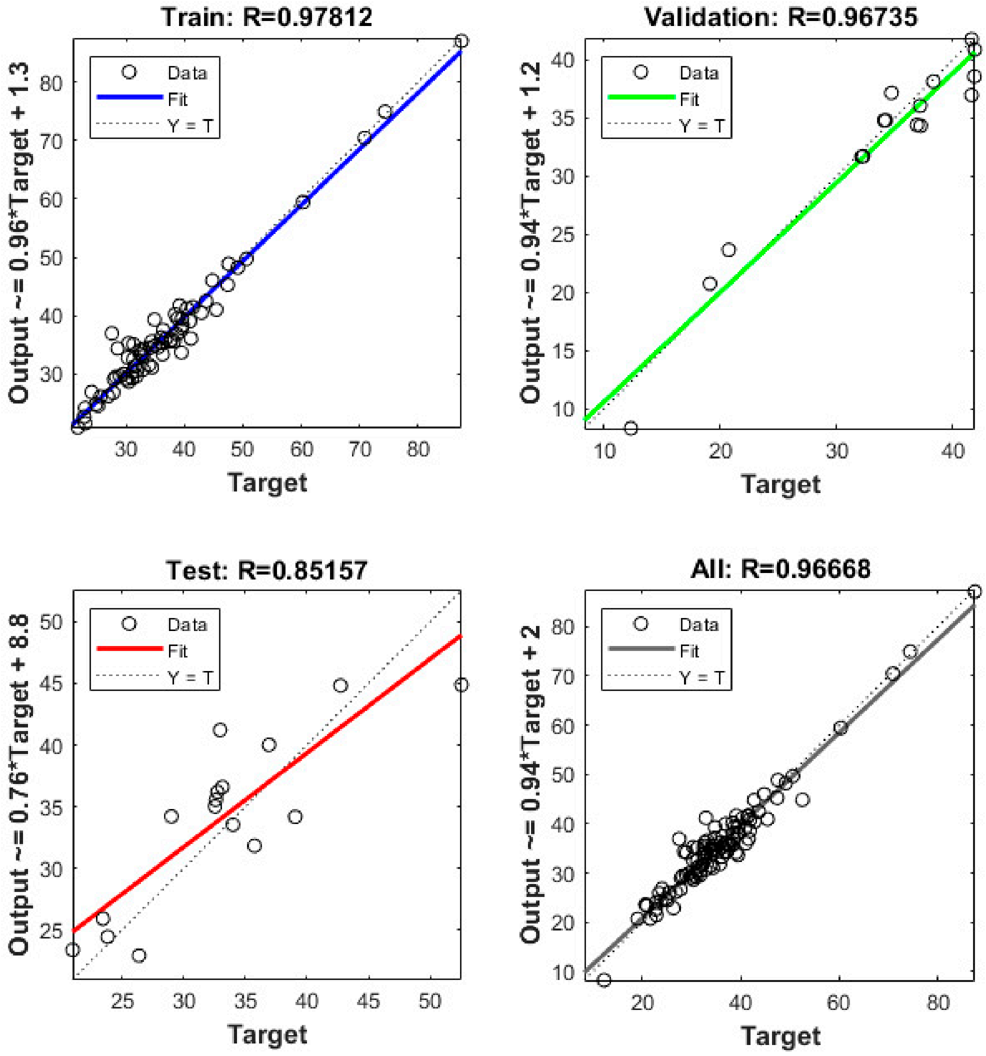

Figure S1. Regression plots for Total fat mass (ANN model)

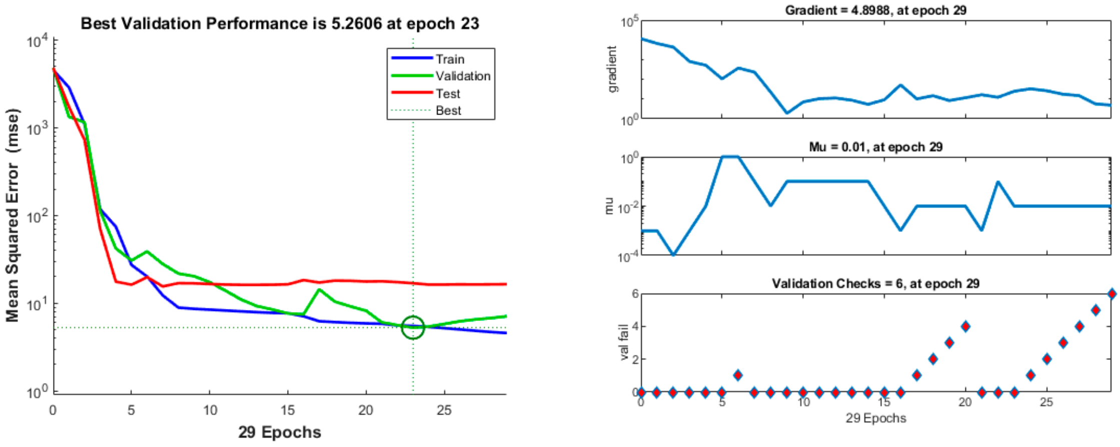

Figure S2. Performance plot (left) and training state plot (right)
for Total fat mass (ANN model)

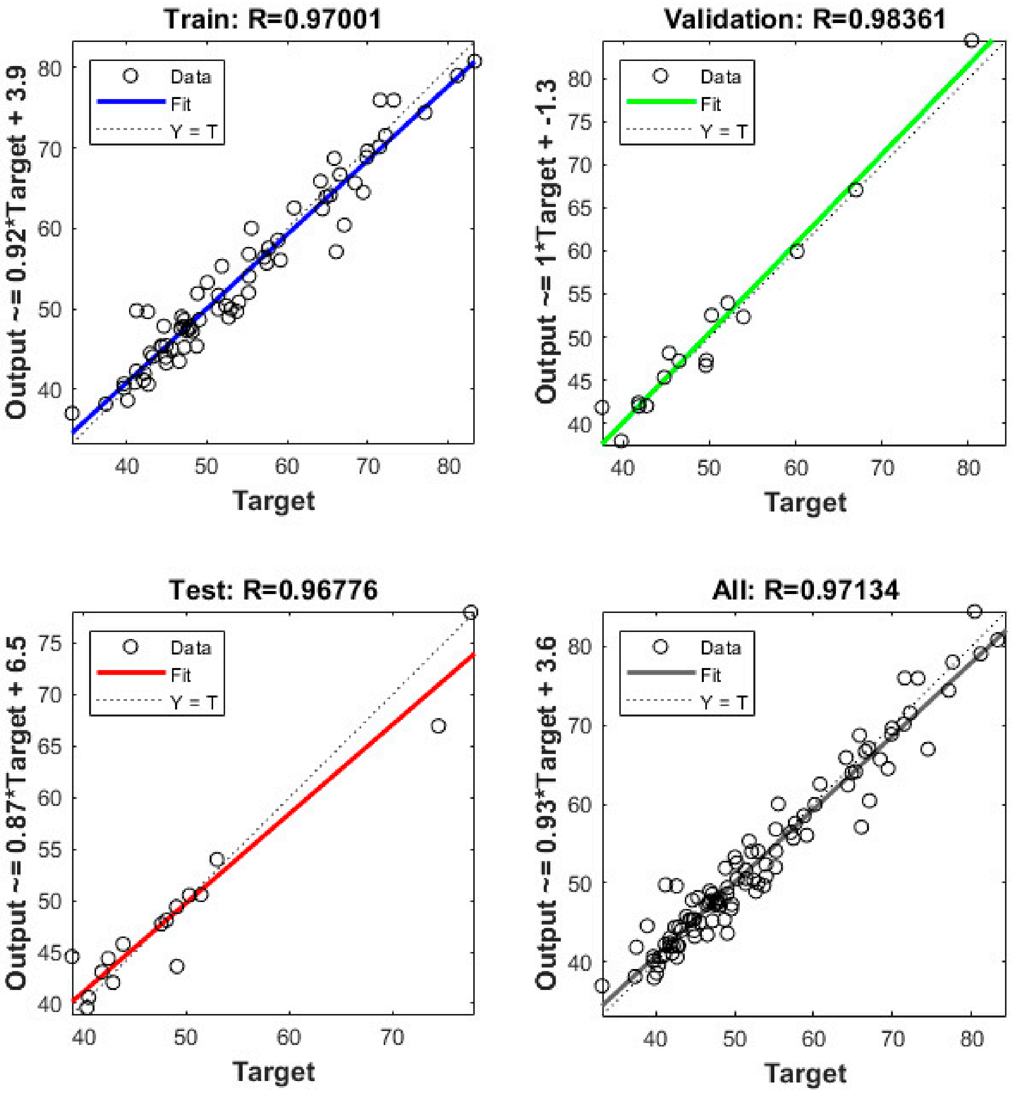

Figure S3. Regression plots for Total fat-free mass (ANN model)

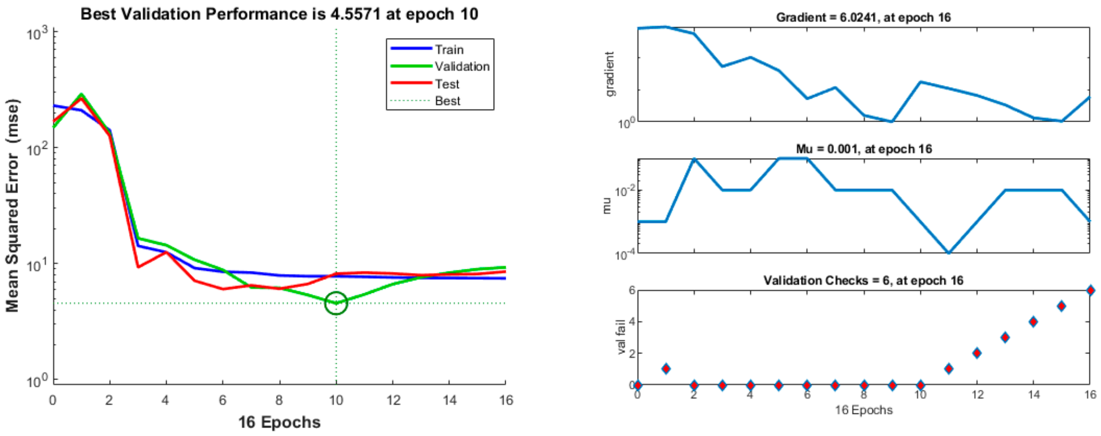

Figure S4. Performance plot (left) and training state plot (right)
for Total fat-free mass (ANN model)

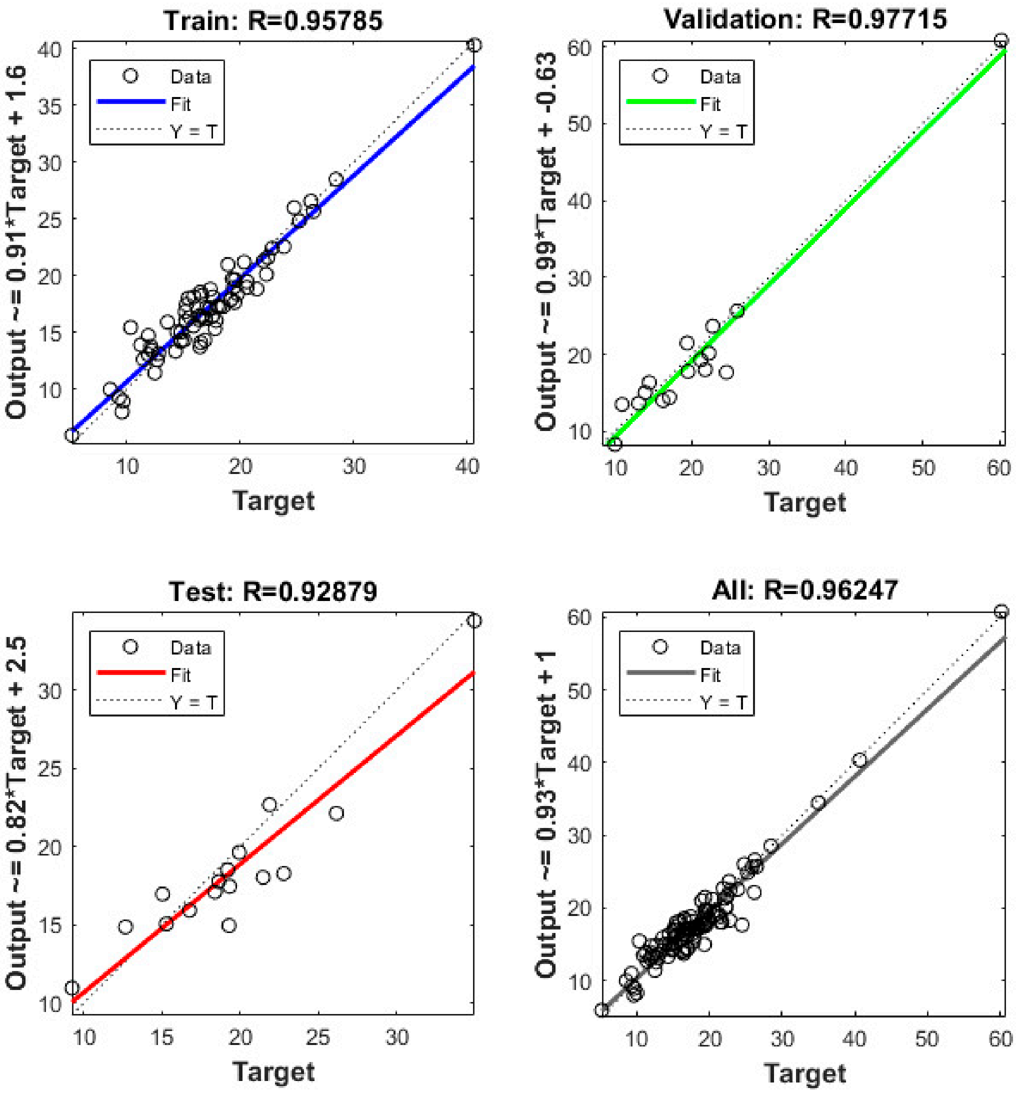

Figure S5. Regression plots for Trunk fat mass (ANN model)

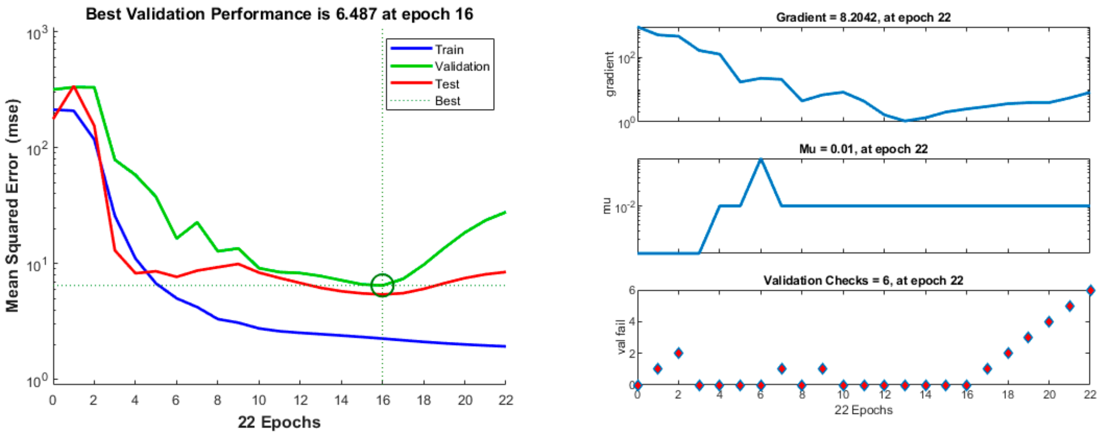

Figure S6. Performance plot (left) and training state plot (right)
for Trunk fat mass (ANN model)

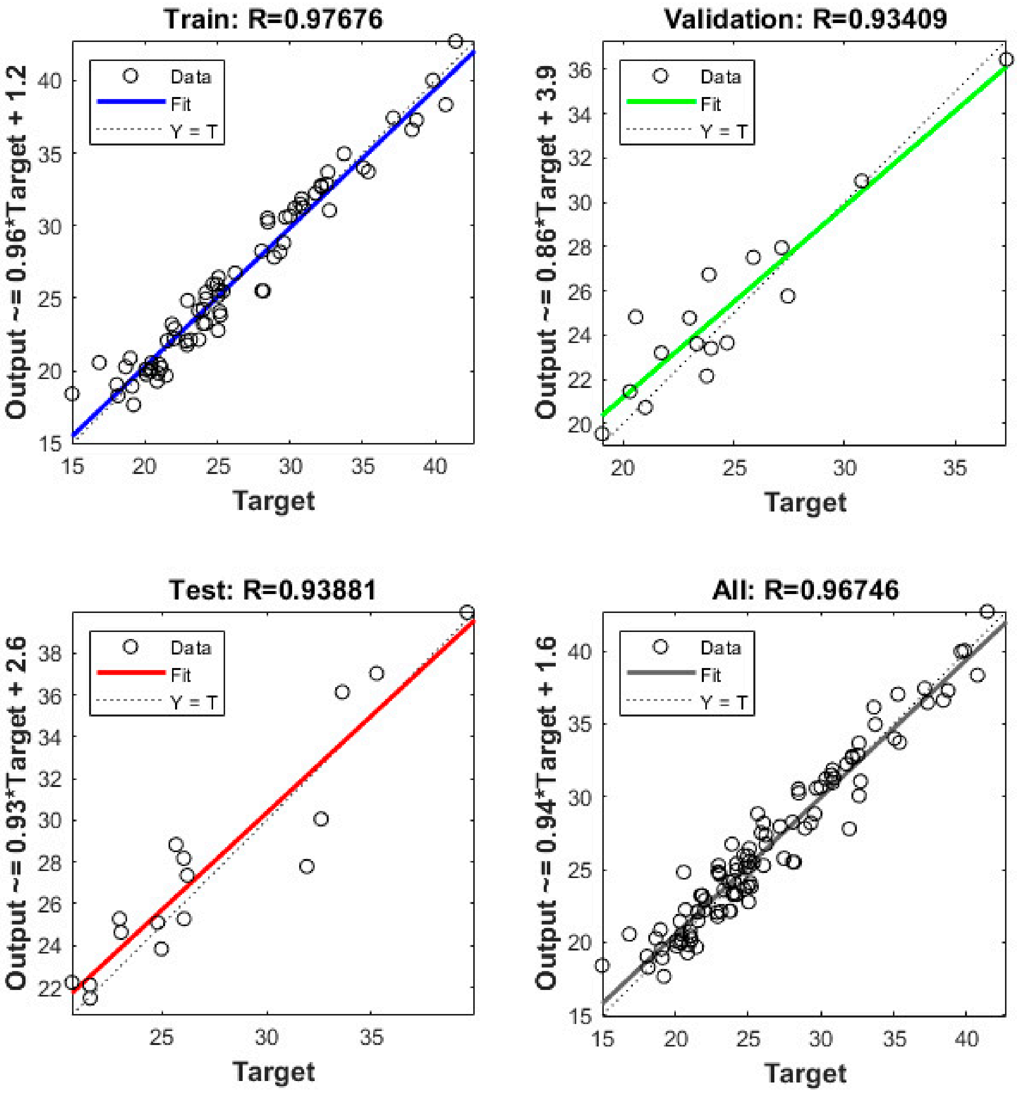

Figure S7. Regression plots for Trunk fat-free mass (ANN model)

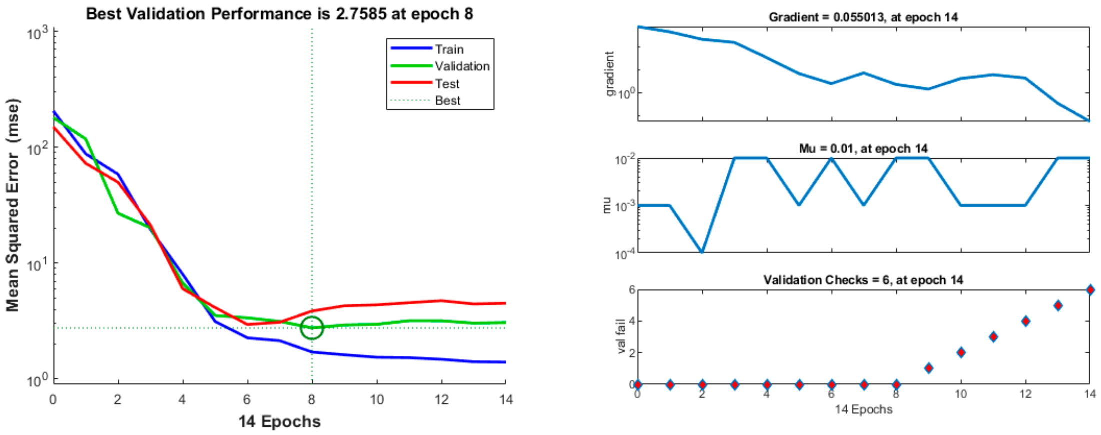

Figure S8. Performance plot (left) and training state plot (right)
for Trunk fat-free mass (ANN model)

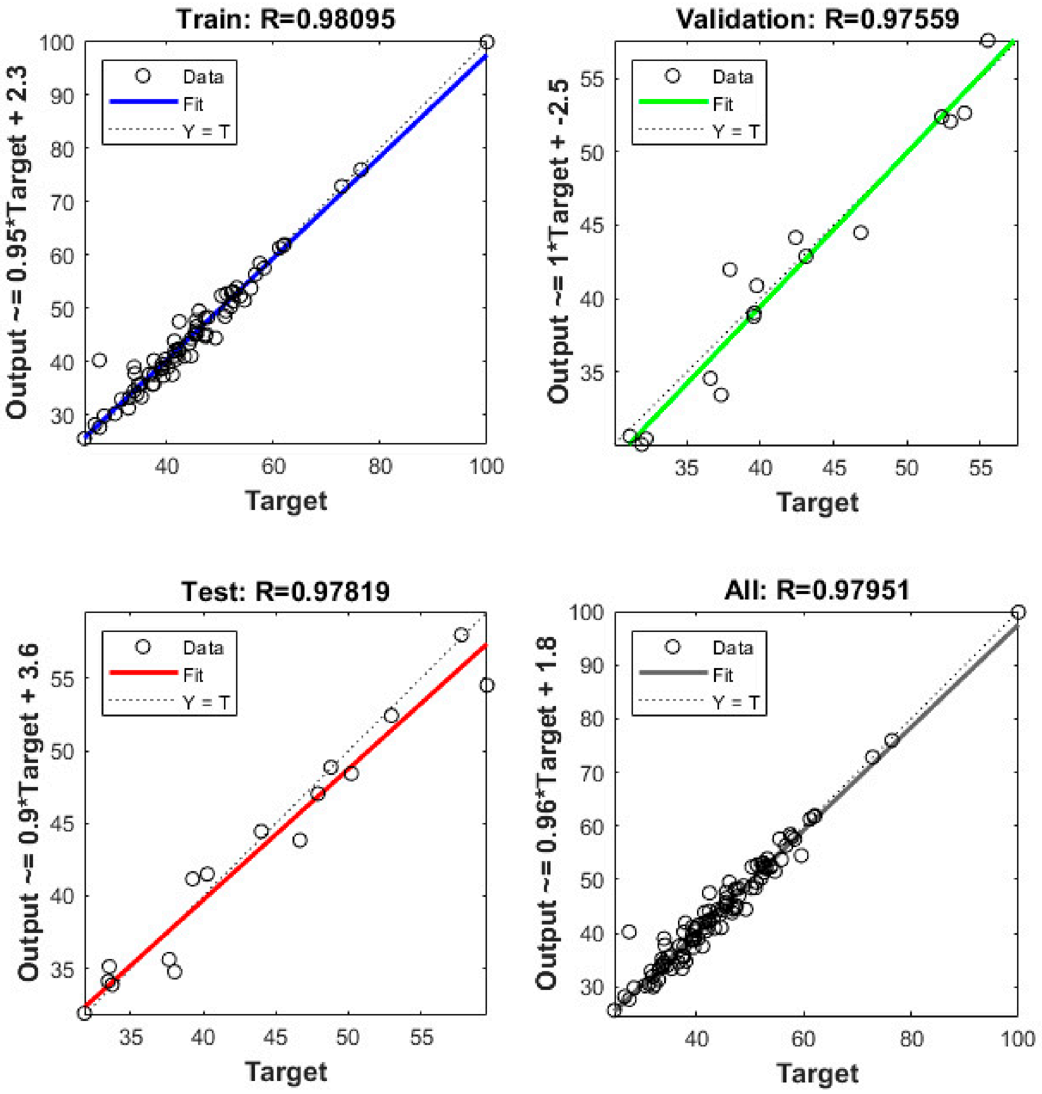

Figure S9. Regression plots for Trunk total mass (ANN model)

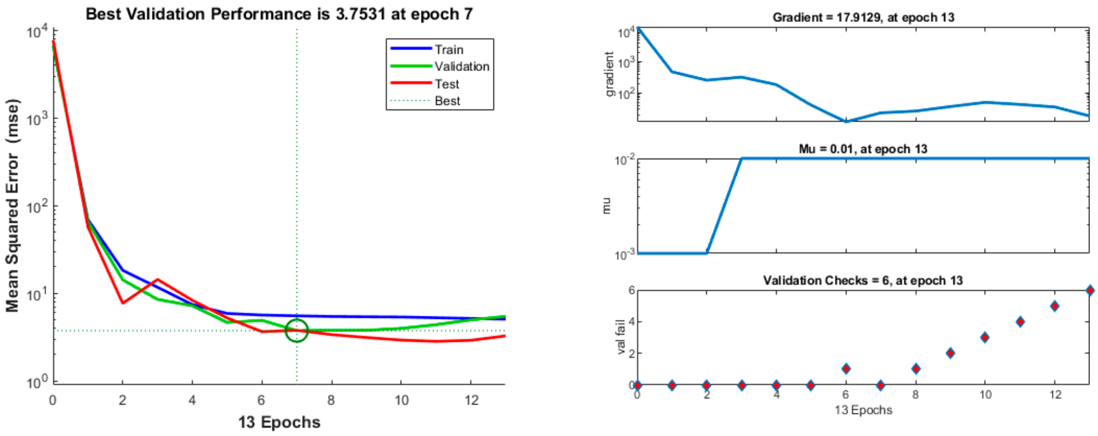

Figure S10. Performance plot (left) and training state plot (right) for Trunk total mass (ANN model)
